# Multi-Omics Analysis Reveals Distinct Lipid Remodelling and Mitochondrial Stress in SH-SY5Y Cells Modelling Parkinson’s Disease

**DOI:** 10.3390/metabo15120781

**Published:** 2025-12-04

**Authors:** Shu Wang, Zhen Ni, Gaoge Wang, Jingzheng Zhang, Yunfu Tan, Enliang Hong, Yunting Wang, Huan Chen, Hongwei Hou, Qingyuan Hu

**Affiliations:** 1 Hefei Institutes of Physical Science, Chinese Academy of Sciences, Hefei 230031, China; 2 Science Island Branch, University of Science and Technology of China, Hefei 230026, China; 3 Beijing Life Science Academy, Beijing 102209, China; 4 China National Tobacco Quality Supervision & Test Centre, Zhengzhou 450000, China

**Keywords:** lipidomics, glycerophospholipids, Parkinson’s disease, mitochondrial stress, multi-omics

## Abstract

**Background**: Neurotoxin-based in vitro models are commonly used to replicate the mitochondrial dysfunction and oxidative stress associated with Parkinson’s disease (PD). While these models reproduce similar hallmark features of PD pathology, their capacity to capture lipid dysregulation remains less well defined. In particular, it is unclear whether different neurotoxins induce distinct glycerophospholipid (GPL) alterations that reflect upstream mechanisms driving mitochondrial impairment. **Methods**: We conducted a comparative multi-omics analysis in SH-SY5Y cells treated with either 6-hydroxydopamine (6-OHDA) or 1-methyl-4-phenylpyridinium (MPP^+^). Lipidomic profiling focused on GPL composition, while transcriptomic changes and organelle stress responses were assessed in parallel, including mitochondrial morphology and lipid droplet accumulation. **Results**: A total of 389 GPL species were identified. MPP^+^ suppressed the expression of mitochondrial genome-encoded respiratory genes and increased polyunsaturated 20:4 GPL species, while selectively depleting odd-chain lipids. In contrast, 6-OHDA activated pathways related to ferroptosis and endoplasmic reticulum stress, along with an accumulation of 20:3 enriched GPLs. In addition, GPL profiles in MPP^+^-treated cells showed a stronger similarity to previously reported alterations in PD patient brain tissue. Despite inducing some shared phenotypes such as lipid droplet accumulation and mitochondrial fragmentation, the two models displayed divergent molecular responses. **Conclusions**: Our findings reveal that MPP^+^ and 6-OHDA drive fundamentally different patterns of GPL remodelling and cellular stress. These results highlight lipid remodelling as a mechanistic indicator of neurotoxin-induced mitochondrial dysfunction and suggest that the MPP^+^ model may provide greater relevance for investigating GPL-related processes in PD.

## 1. Introduction

Parkinson’s disease (PD) is a neurodegenerative condition characterized by the progressive loss of dopamine-producing neurons in the substantia nigra, leading to characteristic motor impairments and widespread cellular disturbances [1,2]. The key pathological features of PD include mitochondrial dysfunction, elevated oxidative stress, and accumulating evidence of lipid metabolic abnormalities [1,2].

To investigate the mechanisms underlying these pathological processes, in vitro models provide valuable platforms under controlled experimental conditions. Among them, the human neuroblastoma SH-SY5Y cell line has emerged as one of the most commonly employed systems for modelling dopaminergic neuron-like phenotypes [3,4,5]. Upon differentiation, SH-SY5Y cells express key neuronal markers and acquire a dopaminergic-like phenotype, making them suitable for modelling PD-associated cellular stress, particularly mitochondrial dysfunction and oxidative injury [3,4,6].

A range of neurotoxins are commonly used to induce PD-like phenotypes in vitro, most of which ultimately converge on mitochondrial dysfunction, a central hallmark of PD pathology. Due to this shared endpoint, these models are often used interchangeably [7,8,9]. Among these, 6-hydroxydopamine (6-OHDA) and 1-methyl-4-phenylpyridinium (MPP^+^) are the most frequently employed [10,11,12,13,14]. While both toxins induce similar downstream phenotypes, such as oxidative stress and impaired bioenergetics, their upstream mechanisms differ significantly [15,16]. These include distinct transcriptional responses, patterns of organelle stress, and divergent lipid remodelling. Whether such differences, particularly in lipid dysregulation, reflect the upstream mechanisms driving mitochondrial dysfunction in PD patients remains unclear.

Lipid dysregulation has recently gained increasing attention as a relevant feature of PD. While ceramides and triacylglycerols have been extensively studied, glycerophospholipids (GPLs) remain less explored, despite evidence of their involvement in PD progression [17,18,19,20]. For instance, the phosphatidylcholine (PC) 20:4_18:2 has been reported several times to be elevated in PD patient [18,19]. Polyunsaturated GPLs are especially prone to peroxidation under conditions of elevated oxidative stress, making them key mediators of lipid-derived toxicity and ferroptosis cell death [21,22]. In addition, mitochondrial-specific GPLs such as cardiolipin are essential for maintaining cristae architecture and sustaining electron transport chain (ETC) function, both of which are profoundly disrupted in PD [23,24].

In this study, we employed the two most widely adopted in vitro models of PD, 6-OHDA- and MPP^+^-treated SH-SY5Y cells, to systematically compare their cellular effects with a particular emphasis on GPL metabolism. By integrating lipidomic and transcriptomic analyses, we delineated the distinct molecular landscapes elicited by each neurotoxin. Our results highlight the limitations inherent in single-model experimental designs and provide mechanistic insight into model-specific alterations in lipid pathways. Notably, the MPP^+^ model showed a higher degree of concordance with GPL changes previously reported in postmortem PD brain tissue.

## 2. Materials and Methods

### 2.1. Cell Culture

SH-SY5Y cells (Procell, Wuhan, China) were grown in DMEM/F12 medium (Thermo Fisher Scientific, Waltham, MA, USA) containing 100 U/mL penicillin, 10% fetal bovine serum, and 10 μg/mL streptomycin. Cultures were kept in a humidified atmosphere at 37 °C with 5% CO_2_. For routine passaging, cells at approximately 90% confluence were dissociated using 0.25% trypsin–EDTA, followed by neutralization with complete medium. Cells were cultured at a 1:4 ratio every three days and were used for experiments within 15 passages to ensure phenotypic stability. Differentiated SH-SY5Y cells were treated with either 6-hydroxydopamine (6-OHDA; Sigma-Aldrich, Darmstadt, Germany, H4381) or 1-methyl-4-phenylpyridinium (MPP^+^; MCE, HY-W008719).

### 2.2. Cell Viability and Functional Assays

#### 2.2.1. Cell Viability Assay

To determine the appropriate concentrations of neurotoxins, SH-SY5Y cells were seeded at a density of 5000 cells per well in 96-well plates and incubated for 24 h. Cells were then treated for an additional 24 h with increasing concentrations of 6-OHDA (0 μM, 10 μM, 25 μM, 50 μM, 100 μM) or MPP^+^ (0 μM, 100 μM, 250 μM, 500 μM, 1000 μM). After treatment, a CCK-8 kit (CK04, Dojindo, Kumamoto, Japan) was applied to each well and incubated at 37 °C for 2 h, and absorbance was read at 450 nm using a Tecan Spark microplate reader (Thermo Scientific, Skanlt Software 7.0.2).

#### 2.2.2. TMRM Assay

Cells were seeded at 3000 cells per well in a 96-well plate and cultured at 37 °C in a humidified incubator with 5% CO_2_ overnight. The next day, cells were treated with either 100 μM 6-OHDA or 500 μM MPP^+^ in the presence of Image-iT™ TMRM Reagent (Invitrogen, I34361, T668). Mitochondrial membrane potential was monitored using a high-content imaging system (RuiFuDi, Shanghai, China) in time-lapse mode, acquiring images every 2 h over a 24 h period. The system’s integrated analysis module was used to quantify the fluorescence intensity.

#### 2.2.3. Seahorse XF Cell Mito Stress Test

Cells were seeded at 3000 cells per well into Seahorse XFe96 culture microplates (Cat. No. 103793-100, Agilent, Santa Clara, CA, USA) at 37 °C overnight. Following 24 h of treatment with 6-OHDA or MPP^+^, mitochondrial respiratory function was evaluated with the Seahorse XF Cell Mito Stress Test Kit (Agilent, Cat. No. 103015-100). Sensor cartridges were hydrated in Seahorse XF Calibrant overnight (20 h) at 37 °C in a non-CO_2_ incubator prior to assay. Immediately before measurement, the cells were rinsed and incubated for 1 h at 37 °C in a non-CO_2_ environment with 180 μL of Seahorse XF Base Medium containing 2 mM L-glutamine, 1 mM pyruvate, and 10 mM glucose (Agilent, Cat. No. 103575-100; 103578-100; 103579-100), adjusted to pH 7.4. Drug injection ports were loaded with the following mitochondrial inhibitors: 1 μM of oligomycin, 2 μM of FCCP, and 0.5 μM of rotenone with 0.5 μM of antimycin A. Oxygen consumption rate (OCR) was recorded using the Seahorse XF96 Analyzer (Agilent Technologies) and analyzed with Wave software (version 2.6.3).

#### 2.2.4. MDA Assay

SH-SY5Y cells were seeded at a density of 1 × 10^6^ cells per 10 cm culture dish and incubated overnight at 37 °C in a humidified incubator with 5% CO_2_. After treatment, the culture medium was removed and cells were lysed using cell lysis buffer (P0013, Beyotime, Shanghai, China). Lysates were centrifuged at 10,000× *g* for 10 min, and the supernatant was collected for further analysis. Malondialdehyde (MDA) levels were determined using a lipid peroxidation MDA assay kit (S0131S, Beyotime), and protein concentrations were quantified with a BCA protein assay kit (PC0020, Solarbio, Beijing, China). Intracellular MDA levels were normalized to total protein content.

### 2.3. Molecular and Imaging Analyses

#### 2.3.1. Confocal Imaging

SH-SY5Y cells were seeded at 10,000 cells per well in 4-well confocal imaging chambers and incubated at 37 °C overnight. A 24 h treatment with either 100 μM 6-OHDA or 500 μM MPP^+^ was applied to cells. Following treatment, culture medium was replaced with one of the following fluorescent probes: LysoBrite™ Green (25154, Cayman Chemical, Ann Arbor, MI, USA) for lysosome staining, PK MITO Orange (PKMO-1, Genvivo Biotech, Nanjing, China) for mitochondria, or BODIPY (121207-31-6, MedChemExpress, Monmouth Junction, NJ, USA) for lipid droplets. Cells were incubated with the respective dye and imaged using a Leica STELLARIS STED super-resolution confocal microscope. The excitation/emission wavelengths were 450/505 nm for lysosomes, 590/610 nm for mitochondria, and 493/503 nm for lipid droplets.

#### 2.3.2. Western Blotting

Cells were lysed using RIPA (Radioimmunoprecipitation Assay) lysis buffer (P0013B, Beyotime, Shanghai, China) with a protease inhibitor cocktail (Beyotime, P1045). Lysates were centrifuged to collect the supernatant. Protein concentrations were determined with the BCA kit (Beyotime, P0010S). Next, 15 μg of protein was separated by SDS–PAGE and transferred onto PVDF membranes. Membranes were blocked in TBST (Tris-buffered saline with 0.1% Tween-20) containing 5% non-fat milk, then incubated at 4 °C overnight with primary antibodies against PGC-1α (66369-1-IG, Proteintech, Wuhan, China), OPA1 (Proteintech, 27733-1-AP), TOM20 (42406S, CST, Danvers, MA, USA), α-tubulin (Proteintech, 66031-1-Ig), DRP1 (Proteintech, 12957-1-AP), MFN2 (Proteintech, 12186-1-AP), MFN1 (Proteintech, 13798-1-AP), GRP78 (HY-P80496, MCE, Monmouth Junction, NJ, USA), and CHOP (ET1703-05, HUABIO, Hangzhou, China). After washes in TBST, membranes were incubated at room temperature with goat anti-rabbit IgG (ab205718, Abcam, Cambridge, UK) or HRP-conjugated goat anti-mouse IgG (Abcam, ab205719). Signals were detected with an ECL kit (Beyotime, P0018AS) and visualized using a Tanon-5200 imaging system. For sequential immunoblotting, membranes were gently stripped using mild stripping buffer (E701-02, Vazyme, Nanjing, China) when target proteins and loading controls shared overlapping molecular weights. Complete removal of primary antibodies was confirmed by the absence of a signal upon secondary antibody-only incubation. Protein bands analysis was performed using ImageJ software (version 1.54k).

### 2.4. Lipidomics, Bioinformatics, and Statistical Analysis

#### 2.4.1. Lipidomic Analysis

Lipids were extracted from approximately 1 × 10^6^ SH-SY5Y cells using a modified Bligh and Dyer protocol [25,26]. Cells were lysed in 750 μL of CH_3_OH:CHCl_3_:H_2_O (6:3:1, *v*/*v*/*v*) and agitated at 1500 rpm for 1 h at 4 °C. Following the addition of 350 μL H_2_O and 250 μL CHCl_3_, centrifugation was performed to separate phases. The lower organic layer was collected, and the aqueous phase was re-extracted with an additional 450 μL CHCl_3_. Organic extracts from both steps were combined and evaporated under vacuum (SpeedVac, OH mode). The aqueous fraction and pellet were dried separately (SpeedVac, H_2_O mode) for later normalization. Lipidomic profiling was performed using a Shimadzu Nexera 20AD HPLC system coupled to a SCIEX QTRAP 6500 PLUS mass spectrometer (LipidALL Technologies, Changzhou, China), following established protocols [27]. Polar lipids were resolved via normal-phase HPLC on a TUP-HB silica column (3 μm, 2.1 mm × 150) using mobile phase A (CH_3_OH:CHCl_3_:NH_4_OH, 10:89.5:0.5, *v*/*v*/*v*) and mobile phase B (NH_4_OH: CH_3_OH:CHCl_3_:H_2_O, 0.5:39:55:5.5, *v*/*v*/*v*/*v*). Multiple reaction monitoring transitions were optimized for the targeted quantification of major glycerophospholipid species. Quantification was achieved by referencing to internal standards including the following: d5-CL72:8(18:2)_4_, C17-SL, d7-PG33:1(18:1_15:0), d7-PI33:1(18:1_15:0), d31-PS(18:1_16:0), d7-PA33:1(18:1_15:0), d7-PE33:1(18:1_15:0), d9-PE36:1p(18:1_18:0p), d9-PC32:0(16:0_16:0), and d9-PC36:1p(18:1_18:0p). To ensure analytical stability, one quality control (QC) sample was injected after every six biological samples, resulting in a total of four QC injections across the entire MS run. QC samples were prepared by pooling equal aliquots from all individual lipid extracts. Final lipid abundances were normalized to each sample to ensure comparability across treatment groups. Normalized datasets were subsequently uploaded to MetaboAnalyst 6.0 for data analysis and visualization.

#### 2.4.2. RNA Sequencing (RNA-Seq)

Cells were collected and sent to BGI Health (Hong Kong, China) Co., Ltd. (https://www.hkbgi.com/, accessed on 17 February 2025) for RNA-seq analysis. Total RNA was extracted and processed into sequencing libraries via poly(A)-based mRNA enrichment, fragmentation, cDNA synthesis, adaptor ligation, and PCR amplification, followed by DNBSEQ sequencing using cPAS technology. Raw reads were quality filtered using SOAPnuke by removing adapter sequences, reads with >20% low-quality bases (Q ≤ 15), or >5% unknown bases. Clean reads were mapped to the human reference genome (GRCh38/hg38) with HISAT2, and transcript abundance was quantified using RSEM. DEGs (Differentially expressed genes) were determined in DESeq2 (v1.34.0) based on log_2_ fold change and q-value criteria. Kyoto Encyclopaedia of Genes and Genomes (KEGG) and Gene Ontology (GO) pathway enrichment were analyzed via hypergeometric testing, and significantly enriched terms were identified with *q* ≤ 0.05. All downstream transcriptomic analyses, including DEG identification, clustering, heatmap and volcano plot generation, and functional enrichment, were performed using the Dr. Tom Multi-omics Data Mining platform (https://biosys.bgi.com, accessed on 24 March 2025).

#### 2.4.3. Statistical Analysis

Statistical analyses were performed using GraphPad Prism 8.0. Group comparisons were conducted using one-way ANOVA followed by Dunnett’s multiple comparisons test. A *p*-value < 0.05 was considered statistically significant.

## 3. Results

### 3.1. MPP^+^ Induces More Pronounced Mitochondrial Dysfunction than 6-OHDA

**Figure 1:**
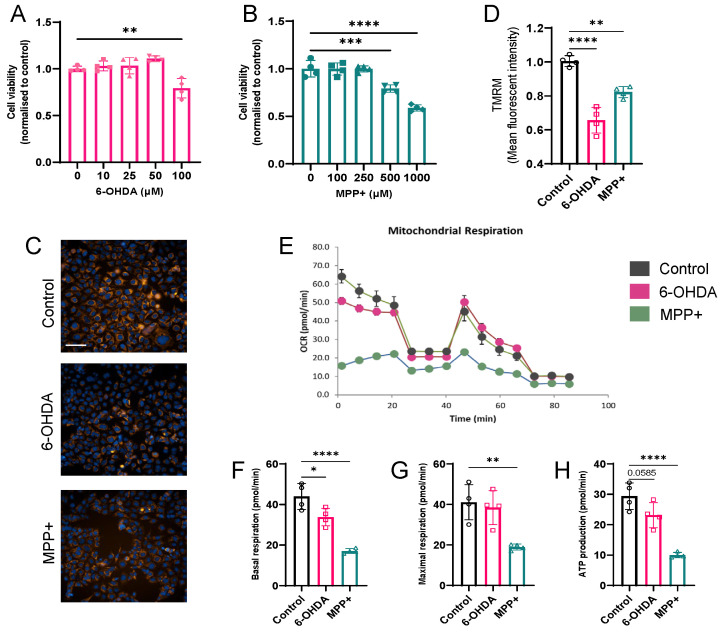
Effects of 6-OHDA and MPP^+^ on mitochondrial function in SH-SY5Y cells. (**A**,**B**) Cell viability in response to different concentrations of 6-OHDA (**A**) and MPP^+^ (**B**) measured after 24 h of treatment. (**C**) Representative high-content imaging of mitochondrial membrane potential following 24 h exposure of neurotoxins (100 μM 6-OHDA or 500 μM MPP^+^). Mitochondria were stained with TMRM (orange); nuclei with Hoechst 33342 (blue). Scale bar: 100 μm. (**D**) Quantification of mitochondrial membrane potential, normalized to control. (**E**) OCR profiles measured using a Seahorse XF analyser. (**F**–**H**) Quantification of basal respiration (**F**), maximal respiration (**G**), and ATP production (**H**) across treatment groups. Data represent four independent experiments. Data are shown as mean ± SD. Statistical comparisons were performed using one-way ANOVA followed by Dunnett’s post hoc test versus control. * *p* < 0.05, ** *p* < 0.01, *** *p* < 0.001, **** *p* < 0.0001.

Mitochondrial dysfunction is a well-established hallmark of PD and represents a primary target of both 6-OHDA and MPP^+^ toxicity. Based on previous research, we first optimized toxin concentrations to achieve comparable levels of cytotoxicity. SH-SY5Y cells treated with 100 µM 6-OHDA or 500 µM MPP^+^ for 24 h exhibited significant reductions in viability (Figure 1A,B), confirming the effective induction of cellular stress at these doses. These concentrations were therefore employed in all subsequent experiments.

To assess mitochondrial membrane potential (ΔΨm) dynamics, we performed 24 h real-time TMRM live cell imaging. MPP^+^ treatment induced an early decline detectable as early as 2 h post treatment, whereas 6-OHDA triggered significant depolarization beginning at approximately 4 h (Figure S1). By 24 h, both toxins led to pronounced ΔΨm collapse (Figure 1C,D).

To further evaluate mitochondrial function, we measured the OCR using the Seahorse XF extracellular flux analyser. Compared with untreated controls, both 6-OHDA and MPP^+^ significantly reduced basal respiration and ATP production, whereas only MPP^+^ further reduced maximal respiratory capacity (Figure 1E–H). Notably, MPP^+^ caused a more profound suppression across all respiratory parameters, consistent with its known mechanism of inhibiting complex I of the ETC [14]. In contrast, 6-OHDA caused a comparatively milder reduction in mitochondrial respiration.

Together, these results indicate that both 100 µM 6-OHDA and 500 µM MPP^+^ impair mitochondrial function, with MPP^+^ eliciting a more severe bioenergetic deficit.

### 3.2. MPP^+^-Induced GPL Profiles Show Greater Concordance with PD Patient Brain than 6-OHDA, but Not with Serum

We performed targeted lipidomic profiling focused on GPLs to investigate neurotoxin-induced lipid remodelling. SH-SY5Y cells were treated with either 100 µM 6-OHDA or 500 µM MPP^+^, and whole-cell lipid extracts were subjected to HPLC/MS-based analysis. Data quality was confirmed by high reproducibility among QC samples (R > 0.99) and low instrument variability (RSD = 7.8%) (Figure S2). A total of 389 lipids were identified (Table S1). Principal component analysis revealed a clear separation between the two treatment groups, indicating distinct patterns of GPLs remodelling induced by each neurotoxin (Figure S2).

**Figure 2:**
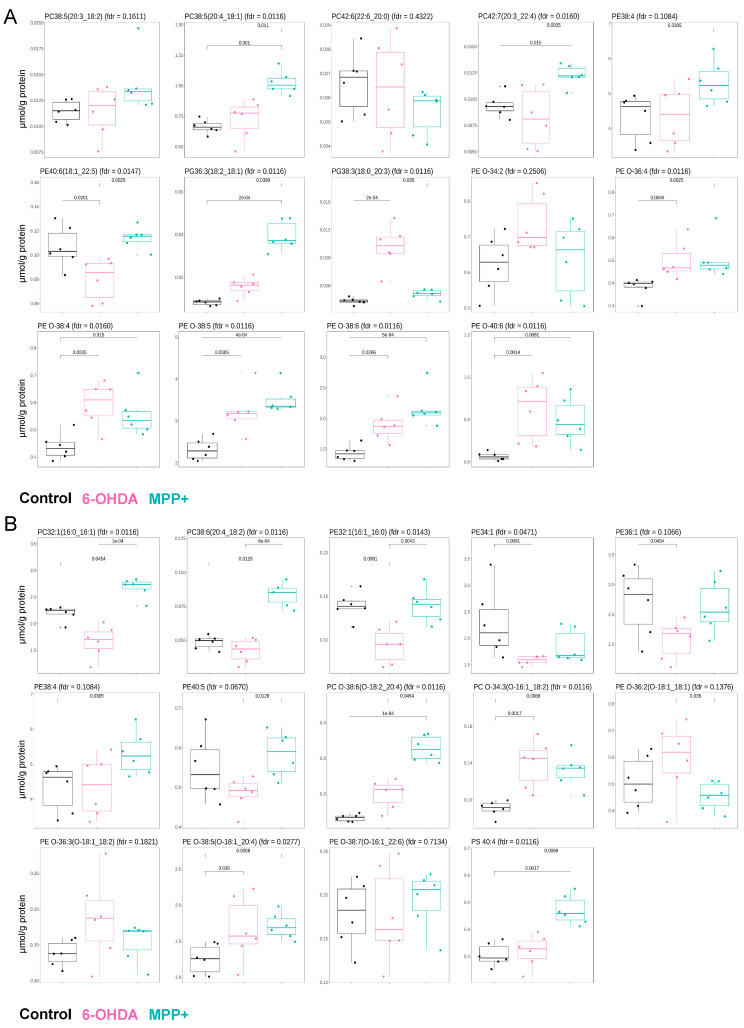
Comparison of specific GPL alterations in SH-SY5Y cells treated with 6-OHDA or MPP^+^. (**A**) Relative abundance of 14 glycerophospholipid (GPL) species previously reported to be significantly or marginally upregulated in the primary motor cortexes of PD patients. (**B**) Relative abundance of 14 GPL species previously identified as significantly altered in the serum of PD patients. Data are presented as mean ± SD (*n* = 6 per group). Neurotoxin concentrations: 100 μM 6-OHDA or 500 μM MPP^+^. Statistical analysis was performed using one-way ANOVA followed by Dunnett’s post hoc test. Adjusted *p* or fdr *q* values are indicated.

We first focused on GPL species previously reported to be altered in clinical samples from PD patients. A recent lipidomic study of postmortem primary motor cortexes from 40 individuals with PD identified eight GPLs significantly and consistently upregulated (*p* < 0.05, *q* < 0.1), including PC 20:0_22:6, PC 20:3_22:4, ether phosphatidylethanolamine (PE O) 16:0_22:6, PE O 16:0_18:2, PE O 18:0_22:6, PE O 16:0_20:4, phosphatidylglycerol (PG) 18:0_20:3, and PG 18:1_18:2. An additional six GPLs were reported to be upregulated with marginal significance (*p* < 0.1): PE 18:1_22:5, PE 18:2_20:2, PC 18:1_20:4, PC 18:2_20:3, PE O 16:0_22:5, and PE O 16:0_22:4 [18]. We examined the abundance of these 14 lipids in our dataset following 6-OHDA or MPP^+^ treatment, compared with untreated controls. In MPP^+^-treated cells, 10 out of 14 lipids showed the same upregulation trend as reported in PD patient brain tissue, while the remaining 4 showed no significant change. In contrast, only 6 out of 14 lipids followed the same upregulation pattern in 6-OHDA-treated cells; 7 remained unchanged, and 1 lipid was downregulated (Figure 2A).

We next assessed concordance with a second dataset, derived from the serum lipidomic profiling of 50 PD patients. In that study, 14 GPL species were identified as altered, PC 32:1, PC 18:2_20:4, PE 16:0_16:1, PE 34:1, PE 36:1, PE 38:4, PE 40:5, PC O 16:1_18:2, PC O 18:2_20:4, PE O 18:1_20:4, PE O 18:1_18:1, phosphatidylserine (PS) 40:4 (*p* < 0.05, *q* < 0.05), PE O 18:1_18:2, and PE O 16:1_22:6 (*p* < 0.05, *q* < 0.15), with PC 32:1 and PC 18:2_20:4 reported as upregulated and the others as downregulated [19]. Among these 14 serum lipids, only 2 matched the same direction of change in MPP^+^-treated cells, and likewise, only 3 in 6-OHDA-treated cells (Figure 2B).

Taken together, these findings suggest that, from the perspective of GPL alterations, the MPP^+^-treated SH-SY5Y model exhibits stronger concordance with clinical PD brain lipid signatures than the 6-OHDA model. However, neither model recapitulates the GPL changes observed in PD patient serum, which may reflect the influence of non-disease factors such as diet, medication, and lifestyle that are not captured in controlled in vitro systems

### 3.3. Acyl Chain Elongation and Unsaturation of Glycerophospholipids in 6-OHDA and MPP^+^ Models May Underlie Increased Susceptibility to Lipid Peroxidation

Long-chain, polyunsaturated phospholipids are highly vulnerable to ROS-induced peroxidation, a key pathological process implicated in dopaminergic neuron loss and ferroptosis. In mouse models, phospholipid peroxides at the plasma membrane have been shown to trigger ferroptosis and drive PD-like motor impairments [22]. Despite a well-established relationship, whether PD models reproduce lipid structural changes that facilitate peroxidation, including longer and more unsaturated acyl chains, has yet to be systematically investigated, thus, we next analyzed these lipid structural parameters.

**Figure 3:**
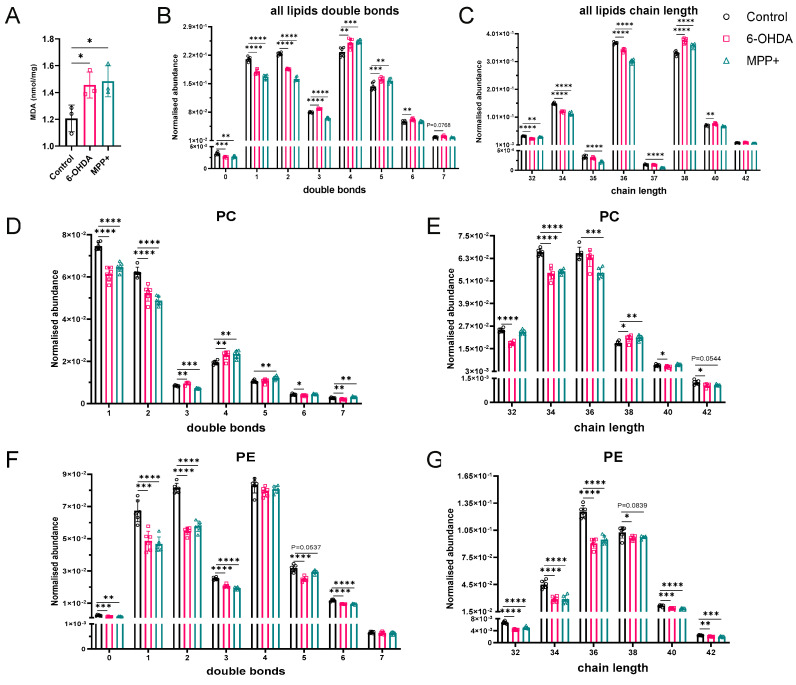
Lipid peroxidation and alterations in double bond number and acyl chain length of GPLs in SH−SY5Y cells treated with 6−OHDA or MPP^+^. (**A**) Quantification of MDA levels as an indicator of lipid peroxidation following 100 μM 6−OHDA or 500 μM MPP^+^ treatment. (**B**,**C**) Global distribution of changes in (**B**) double bond number and (**C**) acyl chain length across all detected GPL species. (**D**–**G**) Subclass-specific alterations in double bond number ((**D**) PC, (**F**) PE) and acyl chain length ((**E**) PC, (**G**) PE) following neurotoxin exposure. Data are presented as mean ± SD (*n* = 6 per group). Statistical significance was assessed using one-way ANOVA followed by Dunnett’s post hoc test. * *p* < 0.05, ** *p* < 0.01, *** *p* < 0.001, **** *p* < 0.0001. Abbreviations: PC, phosphatidylcholine; PE, phosphatidylethanolamine; GPLs, glycerophospholipids.

We first confirmed that both 6-OHDA and MPP^+^ treatments induced lipid peroxidation (Figure 3A). Then, we assessed global patterns of acyl chain unsaturation and length across all identified GPLs. Both models reduced the abundance of saturated and less unsaturated species (0–2 double bonds (DBs)), while increasing highly unsaturated GPLs containing four or five DBs (Figure 3B). Notably, 6-OHDA uniquely elevated GPLs with three, six, and seven DBs, whereas MPP^+^ selectively reduced moderately unsaturated species (DBs = 3), highlighting the divergent remodelling of unsaturation profiles. In terms of chain length, both toxins decreased short-chain GPLs (C32–C36) and increased the abundance of C38 species (Figure 3C). 6-OHDA further increased C40 containing GPLs, whereas C42 species remained unchanged in both models. Interestingly, MPP^+^ selectively depleted odd-chain GPLs (C35, C37), which are typically rare in mammalian cells.

Subclass-specific analyses revealed both shared and distinct patterns of lipid remodelling between the two models. In the PC family, both 6-OHDA and MPP^+^ reduced short-chain, low-unsaturation species and increased those with four DBs and a chain length of C38 (Figure 3D,E). However, 6-OHDA increased PCs with three DBs, whereas MPP^+^ decreased them, an effect reversed with seven double bonds.

PE species were broadly downregulated across all chain lengths and unsaturation levels in both models (Figure 3F,G), suggesting a shared loss of this class, potentially linked to impaired mitochondrial membrane dynamics.

For ether-linked GPLs, both models increased highly unsaturated, long-chain PC Os, while 6-OHDA additionally elevated moderately unsaturated species (Figure S3A,B). In contrast, MPP^+^ only reduced PE Os with two DBs, whereas 6-OHDA increased nearly all PE Os from two to seven DBs and chain lengths up to C42 (Figure S3C,D).

In the phosphatidylinositol (PI) family, MPP^+^ broadly decreased species with one to six DBs, except for a selective increase at four DBs; 6-OHDA induced more targeted increases (Figure S3E,F). Phosphatidic acids (PAs) were generally suppressed in both models, with MPP^+^ showing a uniform decrease and 6-OHDA producing mixed effects (Figure S3G,H). PS species showed model-specific responses: 6-OHDA increased species with three to four DBs, while MPP^+^ elevated those with four to seven (Figure S3I,J). PGs were consistently upregulated in both models at three to five double bonds and chain lengths C34 and C38, representing a shared stress-associated signature (Figure S3K,L).

Together, these findings support our initial hypothesis that PD toxin models promote structural lipid profiles prone to peroxidation, characterized by a general increased chain length and degree of unsaturation. However, the specific remodelling patterns of individual lipid families are model-dependent, underscoring the need for mechanistic precision when selecting in vitro systems to study lipid-driven pathology.

### 3.4. Commonly Used PD Models Induce Opposing GPL Remodelling Patterns

Although both 6-OHDA and MPP^+^ induced a general shift toward longer and more unsaturated glycerophospholipids, global lipidomic comparisons revealed that the underlying remodelling patterns differed substantially between the two models.

**Figure 4:**
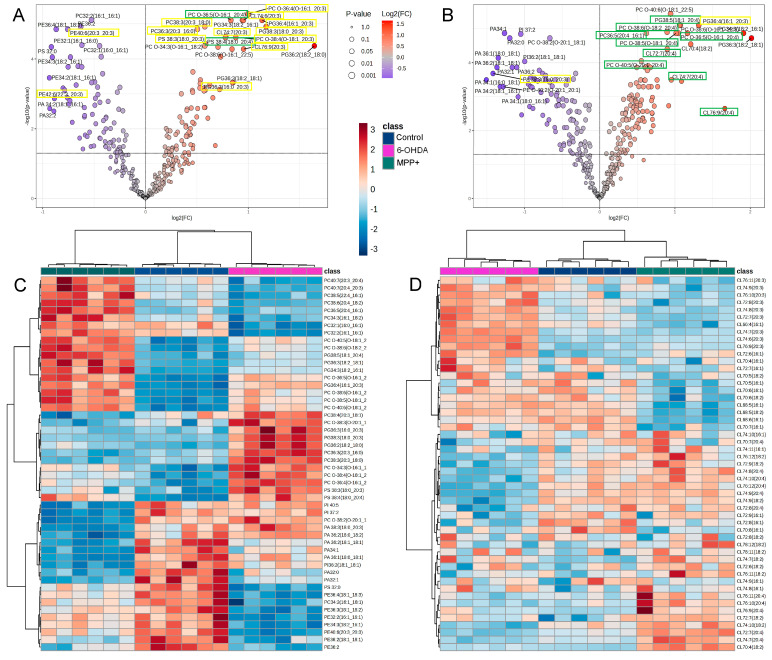
Toxin-specific GPL remodelling patterns in SH−SY5Y cells. (**A**,**B**) Volcano plots showing top 30 significantly altered glycerophospholipid (GPL) species following treatment with (**A**) 6−OHDA or (**B**) MPP^+^, relative to untreated control (*n*=6 per group; *q* < 0.05, fold change > 1). (**C**) Heatmap of the top 50 most significantly altered GPL species across all treatment conditions. (**D**) Heatmap of 51 detected cardiolipin (CL) species. Neurotoxin concentrations: 100 μM 6−OHDA or 500 μM MPP^+^. Upregulated lipids are shown in red and downregulated in purple or blue.

To identify the most strongly dysregulated GPLs under each condition, we performed volcano plot analyses and highlighted the top 30 significantly altered species (*q* < 0.05, FC > 1) for 6-OHDA and MPP^+^. Both treatments markedly upregulated several polyunsaturated PC Os, with PC O 34:3, PC O 36:4, and PC O 36:5 elevated in the 6-OHDA group (Figure 4A), while PC O 38:5, PC O 38:6, and PC O 40:6 were increased following MPP^+^ exposure (Figure 4B). Several PG species, including PG 36:4, PG 36:3 (18:2_18:1), and PG 34:3, were consistently upregulated in both models (Figure 4A,B). In contrast, 6-OHDA treatment resulted in a broad suppression of PE species, particularly those containing saturated and monounsaturated acyl chains (Figure 4A), while PA species were selectively reduced in the MPP^+^ group (Figure 4B).

Strikingly, lipid species enriched in 20:3 or 20:4 side chains showed model-specific enrichment: 11 of the 18 upregulated lipids in the 6-OHDA group contained 20:3, whereas 9 of the 15 upregulated species in the MPP^+^ group contained 20:4 (Figure 4A,B). These included species from the PC, PC O, PG, PS, and CL subclasses, suggesting the presence of toxin-specific acyl chain signatures.

Unsupervised clustering of the 50 most variable GPLs further revealed striking differences between the two models (Figure 4C). While some lipid species showed opposing trends between 6-OHDA and MPP^+^, others were selectively altered in only one condition. Notably, even within the same lipid subclass, for example, PCs, certain species were upregulated exclusively by MPP^+^, others exclusively by 6-OHDA, and some showed opposite trends depending on the treatment (Figure 4C). These findings underscore not just subclass-level divergence, but also toxin-specific remodelling at the level of individual lipid species.

Cardiolipins (CLs), the mitochondria-specific class of GPLs essential for maintaining mitochondrial membrane integrity and respiratory chain activity, also displayed distinct remodelling patterns. Notably, a recent study profiling sex-dependent lipid changes in PD brains identified CLs among the top most perturbed lipid classes [18]. In our dataset, 51 CL species were quantified across both models. Heatmap clustering revealed toxin-specific remodelling: 6-OHDA induced the selective accumulation of CLs containing 20:3 acyl chains, particularly those with total carbon numbers of 72, 74, or 76 (Figure 4D). In contrast, MPP^+^ treatment led to the upregulation of CLs incorporating 16:1, 18:2, or 20:4 chains (Figure 4D).

Together, these results demonstrate that widely used PD neurotoxin models induce distinct and, in some cases, opposing patterns of GPL remodelling. Specifically, 6-OHDA favoured the accumulation of lipids containing 20:3 chains, whereas MPP^+^ showed a preference for those enriched in 20:4. These toxin-specific lipid signatures likely reflect differential mechanisms of mitochondrial stress and membrane remodelling, with important implications for model selection in lipid focused PD studies.

### 3.5. Transcriptomic Differences May Underlie Divergent GPL Remodelling in Different PD Models

To investigate the molecular basis underlying the divergent GPL remodelling patterns observed in the 6-OHDA and MPP^+^ models, we next performed transcriptomic profiling of SH-SY5Y cells treated with either toxin. Despite converging on common PD-related stress responses, the two models exhibited strikingly different transcriptional landscapes, with MPP^+^ altering over 6000 genes and 6-OHDA affecting just under 4000 (*q* < 0.05) (Figure S4, Table S2).

**Figure 5:**
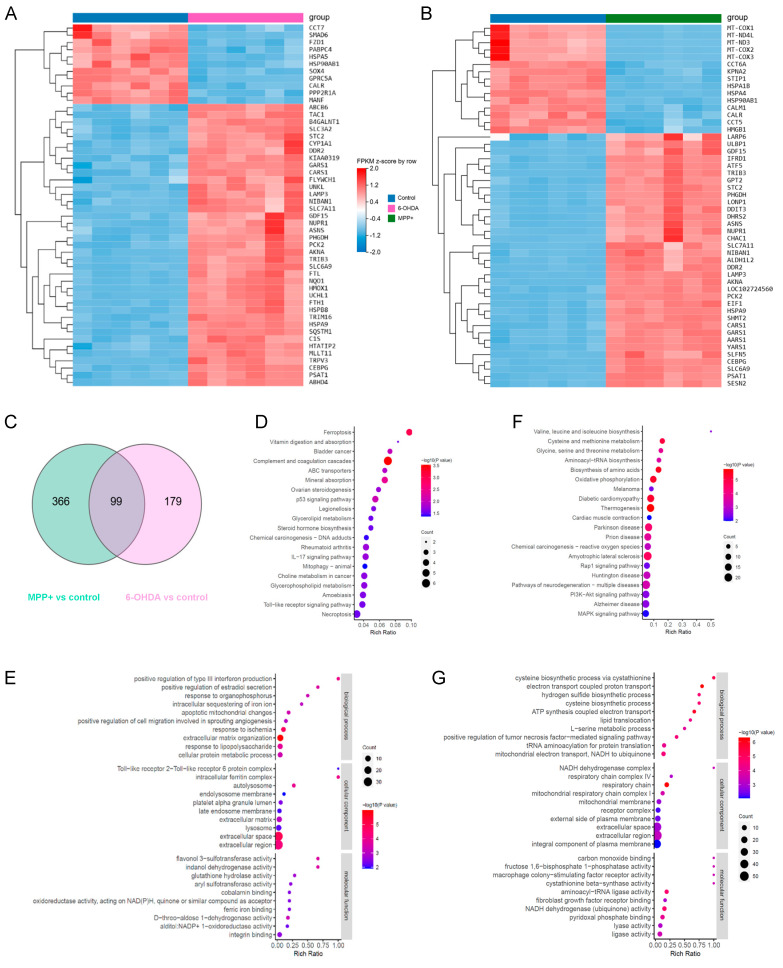
Transcriptomic signatures and pathway enrichment analyses reveal model-specific gene expression programmes in SH-SY5Y cells. (**A**,**B**) Heatmaps displaying hierarchical clustering of the top 50 differentially DEGs ranked by *q*-value in cells treated with (**A**) 100 μM 6-OHDA or (**B**) 500 μM MPP^+^ for 24 h. Each column represents an individual sample (*n* = 6 per group). (**C**) Venn diagram showing the number of DEGs uniquely altered in the 6-OHDA and MPP^+^ treatment groups. DEGs were defined by *q* < 0.05 and |log_2_FC| > 1. (**D**,**F**) KEGG pathway enrichment of uniquely altered DEGs in the (**D**) 6-OHDA and (**F**) MPP^+^ groups, with bubble size indicating gene count and colour representing adjusted *p*-value. (**E**,**G**) GO term enrichment analyses of uniquely altered DEGs in (**E**) 6-OHDA and (**G**) MPP^+^ groups, across three categories: CC, BP, and MF. Abbreviations: DEGs, differentially expressed genes; GO, Gene Ontology; KEGG, Kyoto Encyclopaedia of Genes and Genomes; CC, cellular component; BP, biological process; MF, molecular function.

Analysis of the top 50 DEGs ranked by *q*-value revealed both overlapping and model-specific expression patterns, indicating the presence of both shared and distinct molecular mechanisms. A subset of 15 genes, including PHGDH, PSAT1, and ASNS which mediate metabolic adaptation, as well as GDF15, SLC7A11, and TRIB3, linked to stress resilience, were commonly upregulated in both models, while two genes CALR and HSP90AB1 were consistently downregulated, suggesting convergent disruptions in proteostasis regulation (Figure 5A,B). Within the top 50 DEGs, 6-OHDA selectively downregulated ER-resident chaperones such as HSPA5 and MANF, highlighting the disruption of ER proteostasis and survival signalling (Figure 5A). In contrast, MPP^+^ treatment suppressed mitochondrial genome-encoded components of the ETC, including MT-COX1, MT-COX2, MT-COX3, MT-ND3, and MT-ND4L, implicating impaired mitochondrial bioenergetics as a central pathogenic mechanism (Figure 5B).

To further dissect the biological pathways impacted by each model, we next performed KEGG and GO pathway analysis on the DEGs in the two PD models. Both models shared enrichment for oxidative phosphorylation and neurodegeneration-associated pathways (Figure S5, Table S3). However, 6-OHDA uniquely activated immune and inflammatory responses, while MPP^+^ showed enrichment in proteasome activity, autophagy, and cell cycle regulation (Figure S5). GO terms highlighted 6-OHDA’s impact on apoptosis and morphogenesis, in contrast to MPP^+^’s emphasis on DNA damage response and protein turnover (Figure S5). Both models disrupted mitochondrial function and transcriptional regulation, supporting their shared but divergent mechanisms of neurotoxicity and potentially explaining their different impacts on GPL remodelling.

To specifically investigate the model-unique mechanisms potentially responsible for the divergent lipidomic profiles, we extracted genes exclusively dysregulated in each condition, identifying 366 genes specific to the MPP^+^ group and 179 to the 6-OHDA group (Figure 5C, Table S4; *q* < 0.05, log_2_FC > 1). KEGG and GO enrichment analyses of these gene sets revealed distinct molecular programmes. In the 6-OHDA model, unique DEGs were enriched in pathways related to ferroptosis, glycerolipid metabolism, and GPL metabolism (Figure 5D). GO terms further highlighted biological processes such as intracellular iron sequestration, lipopolysaccharide response, and mitochondrial apoptosis (Figure 5E). In contrast, MPP^+^-specific genes showed strong enrichment for mitochondrial bioenergetic pathways, including oxidative phosphorylation and ETC activity (Figure 5F). GO analysis also revealed perturbations in ATP synthesis, mitochondrial membrane integrity, and NADH dehydrogenase complex function (Figure 5G).

Together, these findings suggest that transcriptomic differences between the 6-OHDA and MPP^+^ models contribute to their distinct patterns of GPL remodelling. The 6-OHDA model preferentially activates pathways related to lipid metabolism and ferroptosis, which are both directly associated with lipid peroxidation. In contrast, MPP^+^ primarily impairs mitochondrial energy metabolism and respiratory chain function, which may indirectly influence lipid composition through disrupted bioenergetic homeostasis. These transcriptional signatures provide a mechanistic framework for understanding how distinct molecular drivers shape lipid remodelling in different PD neurotoxin models.

### 3.6. Organelle Stress Phenotypes Reveal Both Shared and Divergent Features in 6-OHDA and MPP^+^ Models

To determine whether the lipidomic differences observed in the 6-OHDA and MPP^+^ models are accompanied by changes at the organelle level, we examined key subcellular compartments involved in lipid metabolism and stress responses. Mitochondria, lysosomes, and lipid droplets (LDs) are highly sensitive to alterations in membrane lipid composition, and their dysfunction often marks the early stages of neurodegenerative pathology [28,29,30]. We therefore assessed the structural integrity and stress-associated phenotypes of these organelles in both PD models using high-resolution imaging and quantitative analysis.

**Figure 6:**
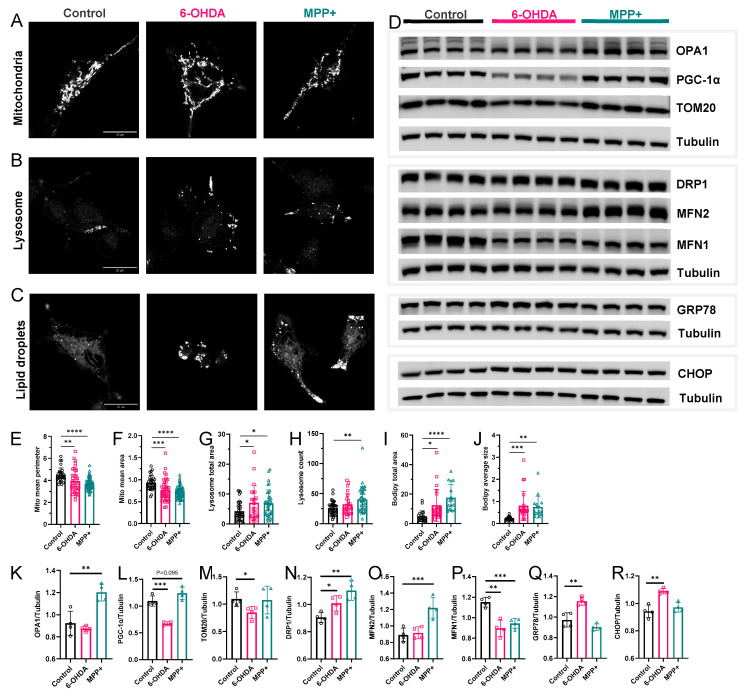
Effects of 6-OHDA and MPP^+^ organelle stress in SH-SY5Y cells. (**A**–**C**) Representative confocal images of (**A**) mitochondria, (**B**) lysosomes, and (**C**) lipid droplets. (**E**–**J**) Quantification of mitochondria (*n* = 30–60 cells), lysosome (*n* = 23–36 cells), or lipid droplets (*n* = 16–23 cells). Scale bar = 20 μm. (**D**) Western blots showing the expression of mitochondrial or ER proteins. (**K**–**R**) Quantification of protein expression levels normalized to tubulin. (*n* = 4). Neurotoxin concentrations: 100 μM 6-OHDA or 500 μM MPP^+^. All data are represented as mean ± SD. Statistical comparisons were conducted using one-way ANOVA followed by Dunnett’s post hoc test. * *p* < 0.05, ** *p* < 0.01, *** *p* < 0.001, **** *p* < 0.0001.

Mitochondria exhibited pronounced fragmentation following treatment with either 6-OHDA or MPP^+^, with a clear transition from elongated tubular networks to punctate structures (Figure 6A). Quantification confirmed significant reductions in average mitochondrial size in both conditions (Figure 6E,F), consistent with increased mitochondrial fission and destabilization. Lysosomes showed marked enlargement and cytoplasmic dispersion, as revealed by LysoBrite™ Green staining (Figure 6B). Both treatments significantly increased total lysosomal area per cell (Figure 6G), while MPP^+^ also elevated the number of lysosomes per cell (Figure 6H). Lipid droplets accumulated substantially in both models, as shown by BODIPY staining (Figure 6C). Quantitative analysis showed significant increases in both LD number and total LD area per cell (Figure 6I,J), indicating altered lipid storage and impaired turnover.

To further characterize these subcellular changes, we examined protein-level markers of organelle stress, focusing on regulators of mitochondrial dynamics, biogenesis, and endoplasmic reticulum (ER) stress. Mitochondrial dynamics were broadly altered in both models. Mitochondrial fission was enhanced in both models, reflected by the increased expression of DRP1 and decreased levels of MFN1 (Figure 6D,N,P), consistent with the fragmented mitochondrial morphology observed by imaging. Interestingly, MPP^+^ uniquely increased OPA1 and MFN2 levels, two fusion-related proteins involved in maintaining cristae structure and inner membrane integrity, suggesting a model-specific response (Figure 6D,K,O). In contrast, TOM20, a marker of outer mitochondrial membrane integrity, was selectively reduced in 6-OHDA-treated cells (Figure 6D,M), indicating mitochondrial import impairment or outer membrane stability.

We also assessed markers of mitochondrial biogenesis and ER stress. PGC-1α, a transcriptional coactivator regulating mitochondrial renewal and antioxidant defence, was reduced in 6-OHDA but modestly increased in MPP^+^ cells (Figure 6D,L), mirroring their distinct transcriptomic profiles. ER stress markers showed a clear model-specific pattern: 6-OHDA treatment led to increased GRP78 and CHOP expression, indicating the activation of the unfolded protein response and pro-apoptotic ER stress signalling, while MPP^+^ had no significant effect on these markers (Figure 6D,Q,R).

Together, these organelle phenotypes underscore that the divergent lipidomic profiles observed in 6-OHDA and MPP^+^ models are accompanied by distinct cellular stress patterns, reinforcing the notion that these models capture different dimensions of PD-related membrane remodelling.

## 4. Discussion

PD is associated with mitochondrial dysfunction, oxidative stress, and increasingly, lipid dysregulation. Although a wide range of in vitro models have been employed to investigate the molecular mechanisms underlying PD, the two most widely used toxins, 6-OHDA and MPP^+^, are often treated as interchangeable tools due to their common endpoint of mitochondrial dysfunction. However, it remains unclear whether these toxins induce distinct upstream stress pathways and how these differences translate into lipid metabolic outcomes relevant to PD pathology. To address this question, we systematically compared the effects of 6-OHDA and MPP^+^ in differentiated SH-SY5Y cells using an integrated multi-omics approach, including GPL-focused lipidomics, RNA-seq, and organelle stress assays. We found that while both toxins triggered GPL remodelling, they did so via distinct acyl chain preferences and cellular pathways. MPP^+^ treatment preferentially enriched 20:4-containing GPLs and recapitulated brain-derived lipid signatures from PD patients, whereas 6-OHDA favoured 20:3-containing species associated with ferroptosis susceptibility. These lipid changes were supported by model-specific transcriptomic responses, including mitochondrial impairment and proteostasis collapse. Our findings highlight the importance of model selection in PD lipidomics research and offer mechanistic insight into how different toxins reproduce distinct aspects of PD-related lipid and mitochondrial pathophysiology.

### 4.1. GPL Signatures Reveal Model-Specific Acyl Chain Remodelling and Stronger Concordance of MPP^+^ with PD Brain Profiles

Benchmarking our lipidomic profiles against previously reported GPL alterations in postmortem PD brain tissue revealed a markedly higher degree of concordance in the MPP^+^ model than in 6-OHDA [18]. Of the 14 brain-derived GPL species consistently elevated in PD patients, 10 were similarly upregulated following MPP^+^ treatment, compared with only 6 in the 6-OHDA model. These included several polyunsaturated PCs and PE Os, lipid species increasingly implicated in mitochondrial membrane integrity and redox vulnerability [31,32]. The closer alignment between MPP^+^-treated cells and patient brain profiles highlights the translational advantage of this model for studying lipid-driven neurodegenerative mechanisms. Notably, this concordance was limited to brain tissue and not observed with serum-derived profiles. This discrepancy likely reflects the multifactorial nature of systemic lipid metabolism in vivo, which is influenced by diet, medications, and metabolic comorbidities. Such confounders are absent in controlled in vitro conditions, rendering serum lipid profiles less applicable for model validation.

Both neurotoxins induced substantial GPL remodelling, favouring species with longer and more unsaturated acyl chains, which are structural traits that increase membrane susceptibility to peroxidation [33,34]. However, their acyl chain preferences diverged significantly: MPP^+^ selectively enriched 20:4 (arachidonic acid)-containing GPLs, known precursors for pro-inflammatory lipid mediators and key participants in oxidative membrane damage, mitochondrial curvature, and bioenergetic collapse [35,36]. In contrast, 6-OHDA increased levels of 20:3 acyl chains, which are omega-6 fatty acid intermediates associated with lipid peroxidation sensitivity and ferroptosis initiation [37,38]. This bias was consistent across multiple GPL subclasses, including PC, PG, CL, and extended to ether-linked species. These compositional shifts are unlikely to be random and may reflect distinct upstream enzymatic and transcriptional programmes.

20:4-enriched phospholipids are regulated by the PLA2 family including PLA2G6, a gene frequently mutated in familial PD [39]. These lipids are key substrates for pro-inflammatory lipid mediator synthesis and membrane curvature modulation, both contributing to mitochondrial stress and energetic collapse in the MPP^+^ model [35,36]. In contrast, 6-OHDA selectively increased 20:3-containing GPLs, likely due to altered elongase or desaturase activity and enhanced turnover through ferroptosis-associated lipid pathways [37,38]. Notably, all these pathways are mechanistically relevant to PD pathology. In addition, a compelling genetic correlation has emerged specifically for 20:3 and 20:4, which uniquely show among polyunsaturated fatty acids concurrent associations with both PD pathogenesis and blood levels [40].

Ether lipids, implicated in modulating oxidative susceptibility, displayed subclass specific alterations in our models [32,41]. Polyunsaturated PC Os were elevated in both models, reflecting a shared adaptation to oxidative stress. However, polyunsaturated PE Os were selectively upregulated only in the 6-OHDA group. Strikingly, when benchmarked against PE O species previously reported to be elevated in PD brains, the MPP^+^ model showed a higher concordance, despite lacking global PE O elevation. This suggests that lipid alterations in MPP^+^ cells may reflect targeted regulatory responses rather than broad lipid stress, further supporting the model’s relevance to PD pathology.

### 4.2. Integrated Transcriptomic and Organelle Stress Responses Support Lipid Remodelling Divergence

The distinct GPL remodelling patterns induced by 6-OHDA and MPP^+^ are further supported by their divergent transcriptomic and subcellular stress responses.

In the 6-OHDA model, the enrichment of pathways involved in ferroptosis, GPL metabolism, and intracellular iron regulation suggest a transcriptional programme directly engaged with lipid peroxidation vulnerability [42]. This mechanistic context is consistent with the selective accumulation of 20:3-containing lipids and PE O species, which are associated with redox imbalance and ferroptosis susceptibility [32,37]. Correspondingly, 6-OHDA-treated cells also showed elevated ER stress markers of GRP78 and CHOP, accompanied by reduced TOM20 expression, pointing to compromised protein folding and mitochondrial import as downstream consequences of lipid dysregulation [43,44,45]. Together, these transcriptomic and organelle changes provide a coherent mechanistic explanation for the observed enrichment of ferroptosis-prone lipid species in 6-OHDA-treated cells.

In contrast, MPP^+^ exposure predominantly impaired mitochondrial oxidative phosphorylation and proteasomal degradation pathways, as reflected by the transcriptomic suppression of ETC components and ATP synthesis machinery [46,47]. This transcriptional profile aligns with the observed lipidomic enrichment of 20:4, which is closely linked to membrane curvature and inflammation-related signalling cascades [35,36]. Notably, despite inducing marked mitochondrial fragmentation at the morphological level, MPP^+^ also triggered a moderate transcriptional upregulation of OPA1, MFN2, and PGC-1α, the key regulators of mitochondrial fusion and biogenesis. Given the 24 h time point examined, this increase may suggest the activation of a compensatory mitochondrial maintenance programme in response to early energetic stress [48,49,50]. Thus, the integration of lipidomic and transcriptomic data reinforces the view that MPP^+^-induced 20:4 enrichment is closely tied to mitochondrial dysfunction and compensatory biogenesis responses.

### 4.3. Limitations and Future Directions

Several limitations should be acknowledged. All experiments were performed with SH-SY5Y cells, which, despite their utility for mechanistic studies, do not fully capture the dopaminergic identity and synaptic features of more physiologically relevant neuronal models. Future work should validate these GPL signatures in advanced in vitro systems such as iPSC-derived dopaminergic neurons and midbrain organoids. Moreover, this study focused on transcriptomic and lipidomic profiling. To better understand how gene-level changes translate into lipid remodelling and organelle stress, the integration of additional omics layers will be useful. Investigating how the perturbation of key enzymes like PLA2G6 influences lipid composition and stress vulnerability within these models may provide mechanistic clarity. Finally, our analyses were limited to a single time point. Evaluating the temporal progression of lipid and stress responses would help distinguish between early adaptive changes and later pathological shifts, offering a more comprehensive view of model-specific dynamics.

## 5. Conclusions

Together, our findings demonstrate that 6-OHDA and MPP^+^ elicit fundamentally distinct patterns of GPL remodelling, underpinned by divergent upstream transcriptional and cellular stress responses. While both models are valuable depending on the research context, our data support the preferential use of MPP^+^ for investigating GPL-driven mechanisms relevant to PD. More broadly, these results highlight the need for mechanism-informed PD model selection, particularly in studies probing lipid metabolism and mitochondrial dysfunction.

## Data Availability

The data in this research are accessible from the corresponding author on inquiry.

## Supplementary Materials

The following supporting information can be downloaded at: https://www.mdpi.com/article/10.3390/metabo15120781/s1. Figure S1. Effects of 6-OHDA and MPP^+^ on mitochondrial membrane potential in SH-SY5Y cells. Figure S2. Quality control assessment of lipidomic profiling. Figure S3. Lipid alterations in double bond number and acyl chain length of GPLs in SH-SY5Y cells treated with 6-OHDA or MPP^+^. Figure S4. Transcriptomic profiling of SH-SY5Y cells exposed to 6-OHDA or MPP^+^ reveals distinct patterns of DEGs. Figure S5. KEGG and GO analyses reveal transcriptomic alterations in 6-OHDA and MPP^+^ induced PD models. Table S1. Raw lipidomic dataset comprising 389 identified glycerophospholipid species. Table S2. Raw transcriptomic dataset containing 6055 DEGs altered by MPP^+^ and 3856 DEGs altered by 6-OHDA. Table S3. DEG lists used for KEGG and GO pathway enrichment analyses in the two PD models. Table S4. Condition-specific DEGs uniquely dysregulated in each model and used for KEGG and GO pathway enrichment in the two PD models.
