# Supplementary material for: Multi-Omics Analysis Reveals Distinct Lipid Remodelling and Mitochondrial Stress in SH-SY5Y Cells Modelling Parkinson’s Disease
Source: Metabolites. 2025 Dec 4;15(12):781. doi: 10.3390/metabo15120781 (PMC12735079; doi:10.3390/metabo15120781)
Supplement: Supplementary file 1 [file metabolites-15-00781-s001.zip › revised_FV_Supplementary Figures.pdf]

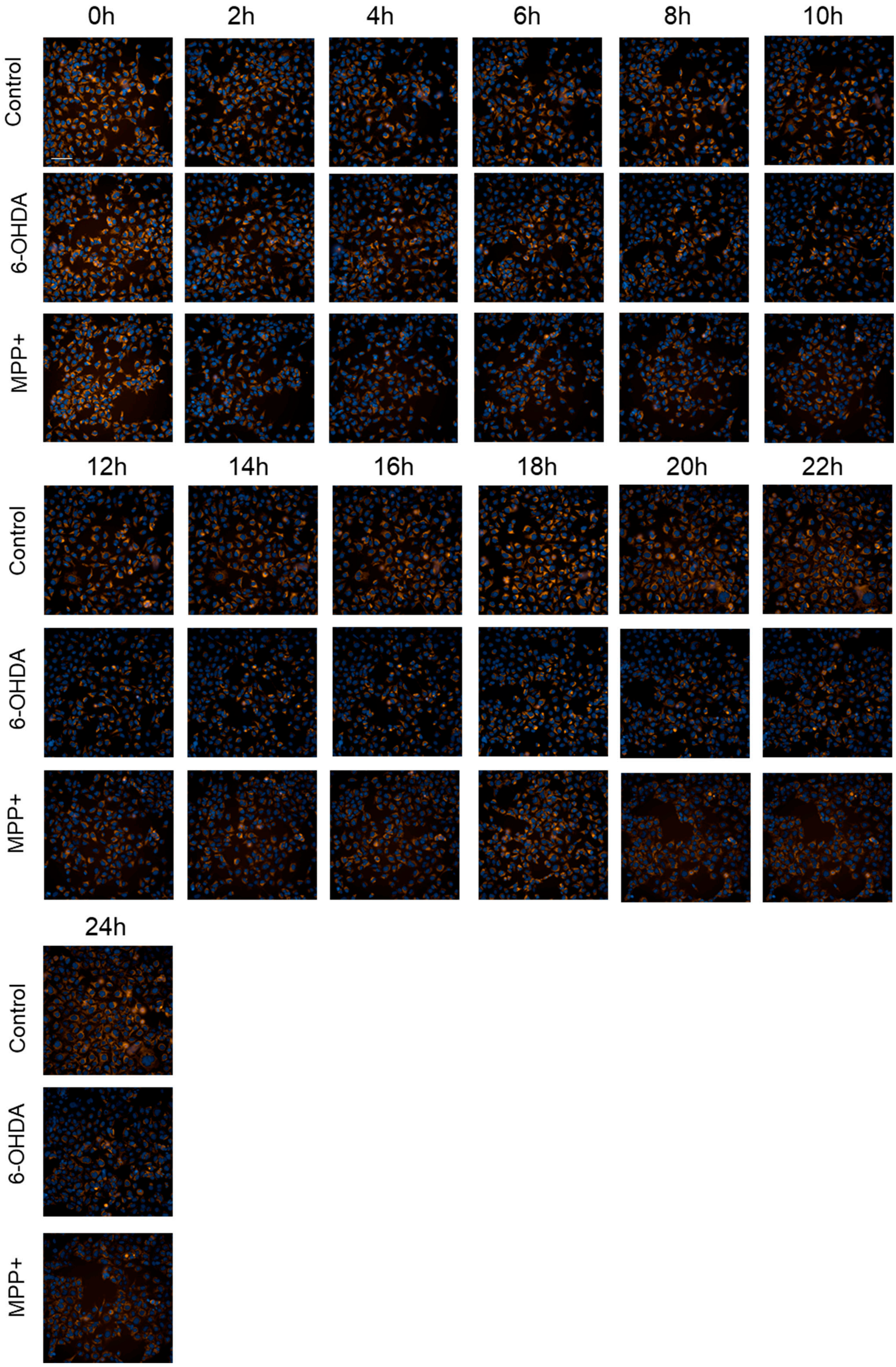

0h
2h
4h
6h
8h
10h
Control
6-OHDA
MPP+
12h
14h
16h
18h
20h
22h
Control
6-OHDA
MPP+
24h
Control
6-OHDA
MPP+

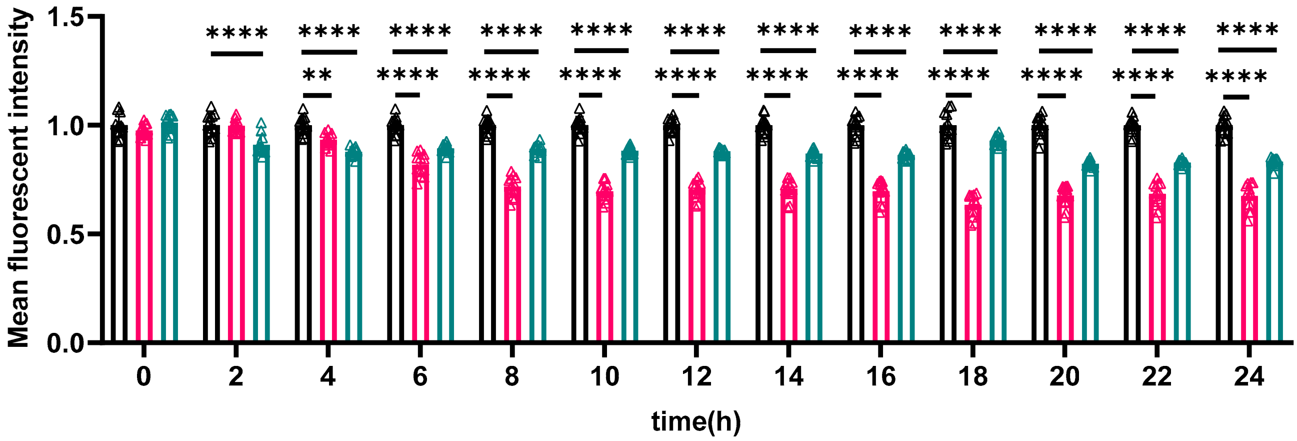

Figure S1. Effects of 6-OHDA and MPP⁺ on mitochondrial membrane potential in SH-SY5Y cells. Representative high-content imaging with quantification of mitochondrial membrane potential following 0-24 h exposure of neurotoxins. Mitochondria were stained with TMRM (orange); nuclei with Hoechst 33342 (blue). Scale bar: 100 μm. Data represent four independent experiments.

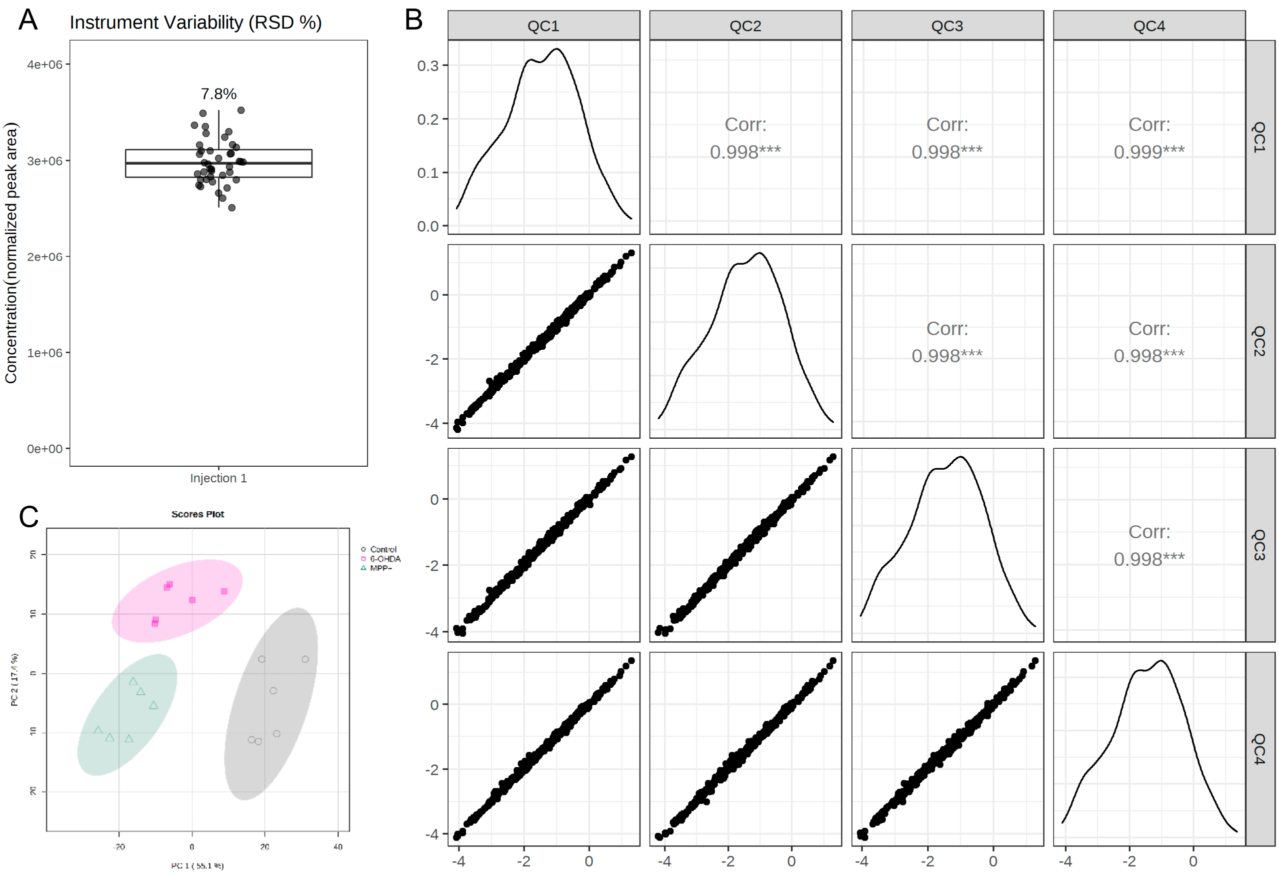

Figure S2. Quality control assessment of lipidomic profiling. A-B. Samples showed low instrument variability with a relative standard deviation (RSD) of 7.8% (A) and strong reproducibility across QCs (Pearson correlation coefficient R > 0.99) (B). **C**. Principal component analysis demonstrated clear separation among experimental groups.

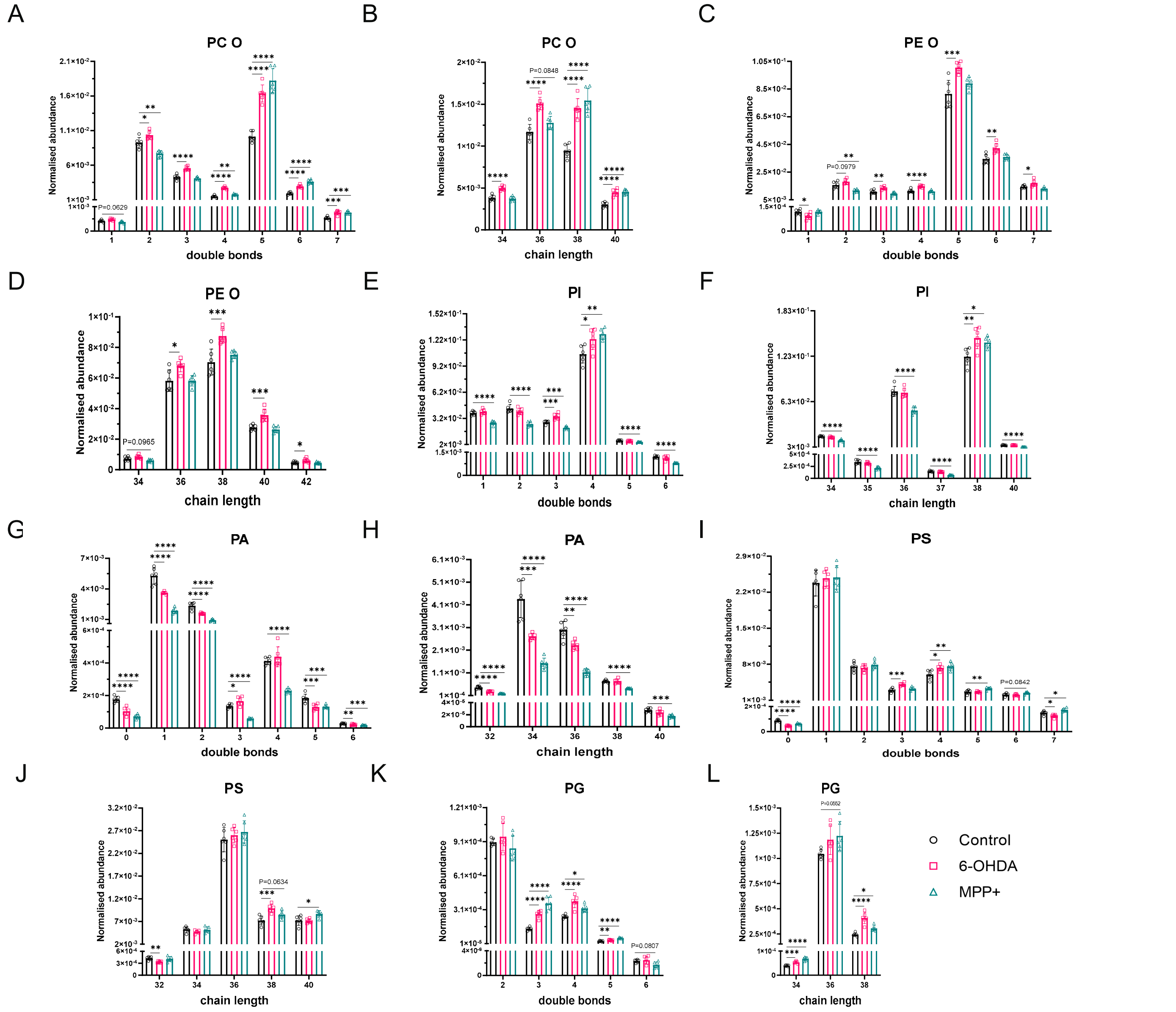

Figure S3. Lipid alterations in double bond number and acyl chain length of GPLs in SH-SY5Y cells treated with 6-OHDA or MPP$^+$. A-L. Subclass specific alterations in double bond number (A. PC-O, C. PE-O, E. PI, G. PA, I. PS, K. PG) and acyl chain length (B. PC-O, D. PE-O, F. PI, H. PA, J. PS, L. PG) following neurotoxin exposure. Data are presented as mean ± SD (n=6 per group). Statistical significance was assessed using one-way ANOVA followed by Dunnett's post hoc test. *p < 0.05, **p < 0.01, ***p < 0.001, ****p < 0.0001. Abbreviations: PC-O and PE-O, ether-linked PC and PE; PI, phosphatidylinositol; PA, phosphatidic acid; PS, phosphatidylserine; PG, phosphatidylglycerol; GPLs, glycerophospholipids.

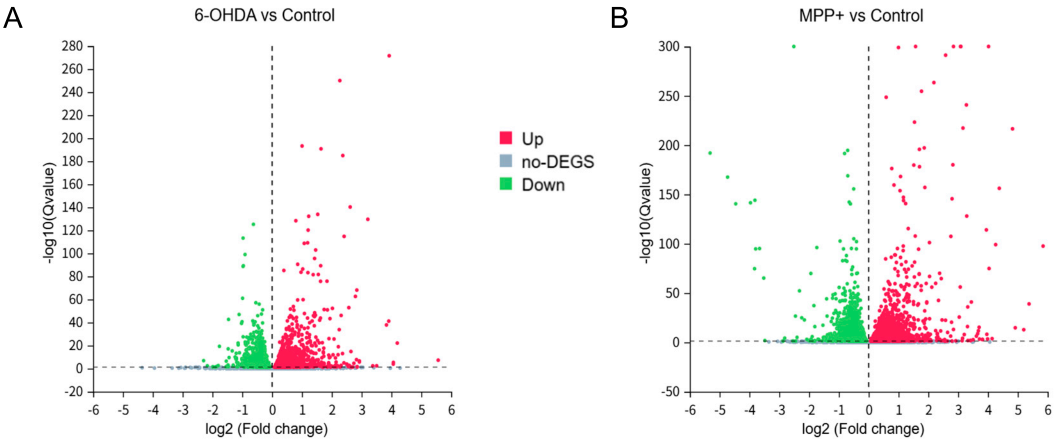

Figure S4. Transcriptomic profiling of SH-SY5Y cells exposed to 6-OHDA or MPP$^+$ reveals distinct patterns of DEGs. A-B. Volcano plots showing DEGs treated with 6-OHDA (A) or MPP$^+$ (B) for 24 h, compared to untreated controls. Significantly upregulated genes are in red, downregulated genes in green ($q < 0.05$).

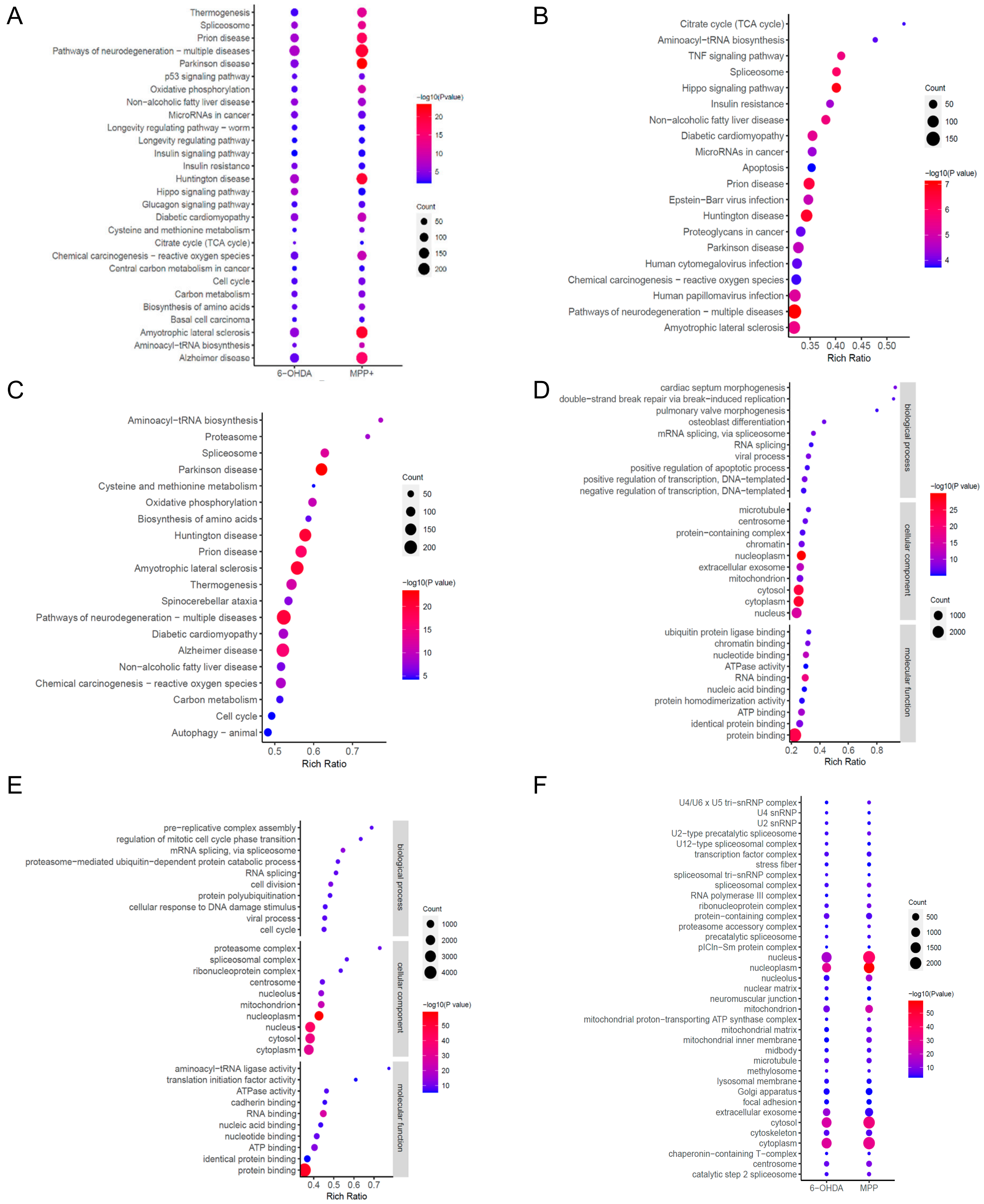

Figure S5. KEGG and GO analyses reveal transcriptomic alterations in 6-OHDA and MPP$^+$ induced PD models. A. Bubble plot illustrating KEGG pathways commonly enriched in the two PD models. **B-C**. Top 20 enriched KEGG pathways identified in the 6-OHDA (B) and MPP$^+$ (C) treatment groups compared to control. **D-E**. Bubble plots showing the top 10 enriched GO terms categorized into CC, BP, and MF in the 6-OHDA (D) or MPP$^+$ (E) models. **F**. Shared GO terms significantly enriched in both neurotoxin models. n=6 per group; DEGs were defined using thresholds of q < 0.05 and $|\log_2 FC| > 0$. Abbreviations: KEGG: Kyoto Encyclopedia of Genes and Genomes; GO: Gene Ontology; CC: Cellular Component; BP: Biological Process; MF: Molecular Function.
